# Palmitate-induced ER stress increases trastuzumab sensitivity in HER2/neu-positive breast cancer cells

**DOI:** 10.1186/s12885-016-2611-8

**Published:** 2016-07-27

**Authors:** Jan Baumann, Jason Wong, Yan Sun, Douglas S. Conklin

**Affiliations:** Department of Biomedical Sciences, Cancer Research Center, State University of New York, University at Albany, Rensselaer, NY 12144 USA

**Keywords:** HER2/neu, ERBB2, Metabolism, Palmitate, Trastuzumab, ER stress, Saturated fat, High fat diet

## Abstract

**Background:**

HER2/neu-positive breast cancer cells have recently been shown to use a unique Warburg-like metabolism for survival and aggressive behavior. These cells exhibit increased fatty acid synthesis and storage compared to normal breast cells or other tumor cells. Disruption of this synthetic process results in apoptosis. Since the addition of physiological doses of exogenous palmitate induces cell death in HER2/neu-positive breast cancer cells, the pathway is likely operating at its limits in these cells. We have studied the response of HER2/neu-positive breast cancer cells to physiological concentrations of exogenous palmitate to identify lipotoxicity-associated consequences of this physiology. Since epidemiological data show that a diet rich in saturated fatty acids is negatively associated with the development of HER2/neu-positive cancer, this cellular physiology may be relevant to the etiology and treatment of the disease. We sought to identify signaling pathways that are regulated by physiological concentrations of exogenous palmitate specifically in HER2/neu-positive breast cancer cells and gain insights into the molecular mechanism and its relevance to disease prevention and treatment.

**Methods:**

Transcriptional profiling was performed to assess programs that are regulated in HER2-normal MCF7 and HER2/neu-positive SKBR3 breast cancer cells in response to exogenous palmitate. Computational analyses were used to define and predict functional relationships and identify networks that are differentially regulated in the two cell lines. These predictions were tested using reporter assays, fluorescence-based high content microscopy, flow cytometry and immunoblotting. Physiological effects were confirmed in HER2/neu-positive BT474 and HCC1569 breast cancer cell lines.

**Results:**

Exogenous palmitate induces functionally distinct transcriptional programs in HER2/neu-positive breast cancer cells. In the lipogenic HER2/neu-positive SKBR3 cell line, palmitate induces a G2 phase cell cycle delay and CHOP-dependent apoptosis as well as a partial activation of the ER stress response network via XBP1 and ATF6. This response appears to be a general feature of HER2/neu-positive breast cancer cells but not cells that overexpress only HER2/neu. Exogenous palmitate reduces HER2 and HER3 protein levels without changes in phosphorylation and sensitizes HER2/neu-positive breast cancer cells to treatment with the HER2-targeted therapy trastuzumab.

**Conclusions:**

Several studies have shown that HER2, FASN and fatty acid synthesis are functionally linked. Exogenous palmitate exerts its toxic effects in part through inducing ER stress, reducing HER2 expression and thereby sensitizing cells to trastuzumab. These data provide further evidence that HER2 signaling and fatty acid metabolism are highly integrated processes that may be important for disease development and progression.

**Electronic supplementary material:**

The online version of this article (doi:10.1186/s12885-016-2611-8) contains supplementary material, which is available to authorized users.

## Background

Approximately 20 % of all breast cancers have increased expression of the ERBB2 (HER2/neu) oncogene [1]. This overexpression is often due to chromosomal amplification and is associated with resistance to chemotherapy, increased recurrence and worse prognosis [2]. Several studies have shown that a number of genes are frequently co-overexpressed or co-amplified along with HER2/neu [1–5]. Several of these co-overexpressed genes have been shown in functional genomics studies to be required for HER2/neu-positive breast cancer cell survival. Since many of these critical genes have roles in fat metabolism and adipogenesis, HER2/neu-positive breast cancer cells possess higher levels of stored triacylglycerides (TAGs) as well as higher levels of saturated fatty acids compared to other cell types [6, 7].

The lipogenic metabolism of HER2/neu-positive breast cancer cells may represent a therapeutic opportunity and have consequences that impact treatment (reviewed recently [8, 9]). The pro-adipogenic transcriptional regulators, PPARγ binding protein (PBP) and RevERBα/NR1D1 cooperatively contribute to increased expression of pro-lipogenic enzymes and a unique, Warburg-like metabolism in HER2/neu-positive breast cancer cells [6]. Overexpression of HER2 itself has also previously been shown to have pro-lipogenic effects translationally increasing protein production of acetyl-CoA carboxylase alpha (ACACA) and fatty acid synthase (FASN) [10]. The result of these genetic changes is the constant production of fatty acids as a means to regenerate reducing equivalents for glycolysis. This occurs through the concerted action of malic enzyme (ME1) and malate dehydrogenase (MDH1), while PBP, NR1D1 and PPARγ orchestrate the sequestration of fatty acids in neutral lipids to avoid lipotoxicity [6, 7]. Since the addition of physiological concentrations of exogenous saturated fatty acids, such as palmitate, induces cell death in HER2/neu-positive breast cancer cells at significantly lower concentrations than in other breast cancer cells or normal human mammary epithelial cells (HMECs) [7], the lipogenic pathway is likely operating at maximum capacity in these cells. This sensitivity of HER2/neu-positive breast cancer cells to palmitate may be related to new epidemiological data that shows that a diet rich in saturated fatty acids is positively associated with the development of HER2/neu-negative disease, but not HER2/neu-positive disease [11]. By modeling the lipotoxicity associated consequences of this physiology we sought to identify signaling pathways that are regulated by physiological concentrations of exogenous palmitate in HER2/neu-positive breast cancer cells and gain insights into the molecular mechanism and its relevance to disease prevention and treatment.

## Methods

### Cell culture and chemicals

Breast cancer cell lines SKBR3, BT474, HCC1569, MCF7 and MCF10A were obtained from the American Type Culture Collection (Manassas, VA, USA) in 2011. Cells were cultured in Dulbecco’s modified Eagle’s medium (DMEM, Hyclone, Logan, UT) supplemented with 10 % fetal bovine serum (Hyclone) at 37 °C and 5 % CO2. MCF10A cells were cultured and infected with pLXSN-neu or vector control retroviruses as previously described [6]. All cell lines were authenticated in March 2016 by the SUNY-Albany Center for Functional Genomics Molecular Core Facility using a short tandem repeat method (Promega GenePrint 10 system). The IRE1 inhibitor STF803010 was obtained from EMD Millipore (Billerica, MA). Trastuzumab was a generous gift from Genentech (San Francisco, CA). Sodium palmitate, MG132 and 4-phenyl butyrate (4-PBA) were obtained from Sigma-Aldrich (St. Louis, MO). Palmitate was solubilized in DMSO or ethanol and diluted in full growth medium (DMEM, 10 % FBS) prior to the treatment of cells. The palmitate concentration was chosen based on the sensitivity profiles of the different HER2/neu-positive breast cancer cell lines. 250 μM palmitate leads to a 70–80 % reduction in viability in SKBR3 cells. According to the literature, fasting FFA concentrations in plasma/serum are in the range of 300–600 μM [12–14] with palmitate representing about one quarter of the total FFAs [15, 16]. However, these concentrations vary extensively based on the analysis method used for quantification [16]. Based on these studies, 250 μM palmitate was deemed to be in the physiological range. All solutions were prepared immediately before usage. For nuclei counts, cells were plated in 96-well plates and allowed to adhere overnight. After treatment and incubation cells were fixed with 2.5 % formaldehyde and nuclei were stained with 1 μg/mL Hoechst 33342 (Life Technologies, Grand Island, NY). Images of cells were acquired using the INCell Analyzer 2200 high-content imaging system and images were analyzed using the INCell Investigator software (GE Healthcare, Piscataway, NJ). For anchorage-independent growth cells were seeded in ultra-low attachment plates in the presence of 250 *μM* palmitate or vehicle control and incubated for 11 days. Viable cells were assessed using the Alamar Blue cell health indicator assay (Life Technologies, Grand Island, NY) [17].

### Microarray analysis

After 24 h of treatment with 250 μM palmitate or vehicle control, cells were harvested by trypsinization and total RNA was extracted using the RNeasy mini kit (Qiagen, Valencia, CA). The quality and the concentrations of total RNA were assessed using the Agilent Bioanalyzer (Agilent Technologies, Santa Clara, CA). Total RNA (100 ng) deemed to be of good quality (RNA integrity number (RIN) greater than 8) was processed according to the standard Affymetrix Whole Transcript Sense Target labeling protocol (Affymetrix, Santa Clara, CA). The fragmented biotin-labeled cDNA from three independent biological replicates was hybridized over 16 h to Affymetrix Gene 1.0 ST arrays and scanned on an Affymetrix Scanner 3000 7G using AGCC software. The resulting CEL files were analyzed for quality using Affymetrix Expression Console software and were imported into GeneSpring GX11.5 (Agilent Technologies) where the data was quantile normalized using PLIER and baseline transformed to the median of the control samples. The probe sets were further filtered to exclude the bottom 20th percentile across all samples as well as probe sets with expression levels with CV *>* 20 % across all replicates in a condition. The resulting entity list was subjected to a t-test with Benjamini-Hochberg FDR correction. The data files have been deposited in Array Express, Accession number: E-MTAB-2601.

### Metabolic assays

For detection of neutral fat stores, cells were stained with 1 μg/ml 4,4-difluoro1,3,5,7,8-pentamethyl-4-bora-3a,4a-diaza-s-indacence (BODIPY 493/503; Life Technologies, Grand Island, NY). Cells were grown in 96-well plates, fixed with 2.5 % formaldehyde, stained with 1 μg/ml BODIPY 493/503 and counterstained for nuclei with 1 μg/mL Hoechst 33342. Cells were imaged using the INCell Analyzer 2200 (GE Healthcare, Piscataway, NJ) high-content imaging system. Images were analyzed using the INCell Investigator software.

### Apoptosis assays

Induction of apoptosis was assessed as described previously using Apo-BrdU staining of fragmented DNA [18]. Treated and control cells were harvested by trypsinization and fixed with 4 % formaldehyde in PBS for 20 min on ice, followed by permeabilization with 70 % EtOH overnight at -20 °C. The 3′-OH ends of fragmented DNA were enzymatically labeled with bromodeoxyuridine triphosphate (Br-dUTP, Phoenix Flow Systems, San Diego, CA), using terminal deoxynucleotidyl transferase (Roche Applied Science) in TdT reaction buffer containing 2.5 mM cobalt chloride (Roche Applied Science) for 1 h at 37 °C. Br-dUTP labeled fragmented DNA was detected using a FITC-conjugated anti-BrdU monoclonal antibody (1:20,BD Pharmingen, San Jose, CA). Cells were counterstained with 5 μg/mL propidium iodide (Sigma-Aldrich) in the presence of 0.015 U/mL RNase (Roche Applied Science) in PBS with 0.1 % Triton X-100 for 30 min at room temperature. Samples were analyzed on a BD LSR II Flow Cytometer (BD Biosciences, San Jose, CA). Percentage of dead cells was assessed by propidium iodide uptake. Cells were trypsinized and incubated in PBS containing 5 μg/mL propidium iodide (Sigma-Aldrich) for 15 min. PI content was determined by flow cytometry. Three independent biological replicates and a minimum of 10,000 events were acquired for each experimental condition. The data were analyzed using the FlowJo software package (Treestar Inc., Ashland, OR).

### Cell cycle analysis

Cell cycle analysis was performed as described previously [19], briefly, the cells were treated for 24 h with palmitate or vehicle control and harvested by trypsinization, followed by 90 % ethanol permeabilization overnight at -20 °C. Permeabilized cells were stained with 5 μg/mL propidium iodide (Sigma-Aldrich) in the presence of 0.015 U/mL RNase (Roche Applied Science) in PBS for 20 min at room temperature. Data were acquired on a BD LSR II Flow Cytometer (BD Biosciences, San Jose, CA) with a minimum of 10,000 events collected for each experimental condition and analyzed using the FlowJo software package (Treestar Inc., Ashland, OR).

### Immunoblotting

Immunoblots were performed using standard protocols. Cells were lysed directly in Laemmli loading buffer, proteins were resolved by SDS-PAGE and transferred to PVDF membranes (EMD Millipore, Billerica, MA). Membranes were blocked in Tris buffered saline (TBS) containing 0.1 % Tween and 5 % non-fat powdered milk (TBS-T, 5 % milk). Primary antibody incubation was performed in TBS-T, 5 % milk overnight at 4 °C. Proteins were visualized using a species-specific HRP-conjugated secondary antibody and the ECL Plus chemifluorescent detection system (Thermo Fisher Scientific Inc., Waltham, MA) on a STORM scanner ((GE Healthcare, Piscataway, NJ). Signal was quantified using ImageJ software ((https://imagej.nih.gov/). The following primary antibodies were used: PERK (#5683, Cell Signaling Technology, Inc., Danvers, MA)(1:1000), phospho-PERK (Thr980, #3179, Cell Signaling) (1:1000), PDI (#3501, Cell Signaling) (1:1000), BiP (#3177, Cell Signaling) (1:1000), eIF2*α* (#5324, Cell Signaling) (1:1000), phospho-eIF2α (Ser51, #3398, Cell Signaling) (1:1000), DDIT3/CHOP (#2895, Cell Signaling) (1:500), HER2 (#4290, Cell Signaling), phospho-HER2 (Tyr1221/1222, #2243, Cell Signaling) (1:1000), HER3 (#4754, Cell Signaling) (1:1000), phospho-HER3 (Tyr1289, #4791, Cell Signaling) (1:1000), EGFR (#4267, Cell Signaling) (1:1000), phospho-EGFR (Tyr1068, #3777, Cell Signaling) (1:1000), GAPDH (#5174, Cell Signaling) (1:15000), *α*-tubulin (MCA78G, AbD Serotec, Oxford, UK) (1:15000). Secondary antibodies: goat-anti-rabbit-HRP (#7074, Cell Signaling) (1:5000), horse-anti-mouse-HRP (#7076, Cell Signaling) (1:5000), goat-anti-rat-HRP (sc-2303, Santa Cruz Biotechnology, Inc., Santa Cruz, CA) (1:15000).

### Statistical and computational analyses

The impact of molecular perturbations (inhibitors, shRNAs) on breast cancer cell phenotypes including cell survival, proliferation, apoptosis resistance, was measured in three independent experiment, from replicate cultures. Means and standard deviations were calculated and results were analyzed for statistical significance using Student’s t-tests for pairwise comparisons or one-way ANOVA with Bonferroni post test, when more than two experimental groups were compared. *P* values < 0.05 were considered statistically significant.

Gene ontology (GO) enrichment analysis was carried out using the DAVID Bioinformatics Resource [20, 21]. Logistic regression analysis for transcription factor motif enrichment was performed using the free web service LRPath (http://lrpath.ncibi.org/). LRpath functionally relates the odds of gene set membership (dependent variable) with the statistical significance of differential expression (independent variable) using logistic regression, and calculates q-values using the FDR method as a measure of statistical significance [22]. The False discovery rate (FDR) is a statistical method when performing multiple comparisons used to control the expected proportion of rejected null hypotheses that were incorrect rejections (“false discoveries”) [23]. The network neighborhood of enriched transcription factors was obtained by querying the STRING database [24].

### Transfections and reporters

For pCAX-XBP1-ΔDBD-venus reporter construct assays [25], cells were seeded in 96-well plates and allowed to adhere overnight before they were transfected using XtremeGene HP (Roche), according to the manufacturer’s instructions. Cells were treated as indicated in the individual experiments, 24 h post-transfection. Expression of the fluorescent protein is indicative of IRE1-mediated XBP1 splicing. The pCAX-XBP1-ΔDBD-venus reporter construct was a generous gift from Dr. Masayuki Miura, University of Tokyo. shRNA-mediated knockdown experiments were carried out using pSM2 constructs obtained from Open Biosystems. Transfections were performed as described for pCAX-XBP1-ΔDBD-venus experiments and cells were treated 48 h post-transfection. Proteasome activity was tested using the ZsProSensor-1 reporter construct which utilizes the ZsGreen1 fluorescent protein coupled to a proteasome-targeting sequence (Clontech, Mountain View, CA). Accumulation of fluorescence in the cell is indicative of decreased proteasome activity.

## Results

### Physiological concentrations of saturated fat induce distinct responses in breast cancer cells

**Fig. 1:**
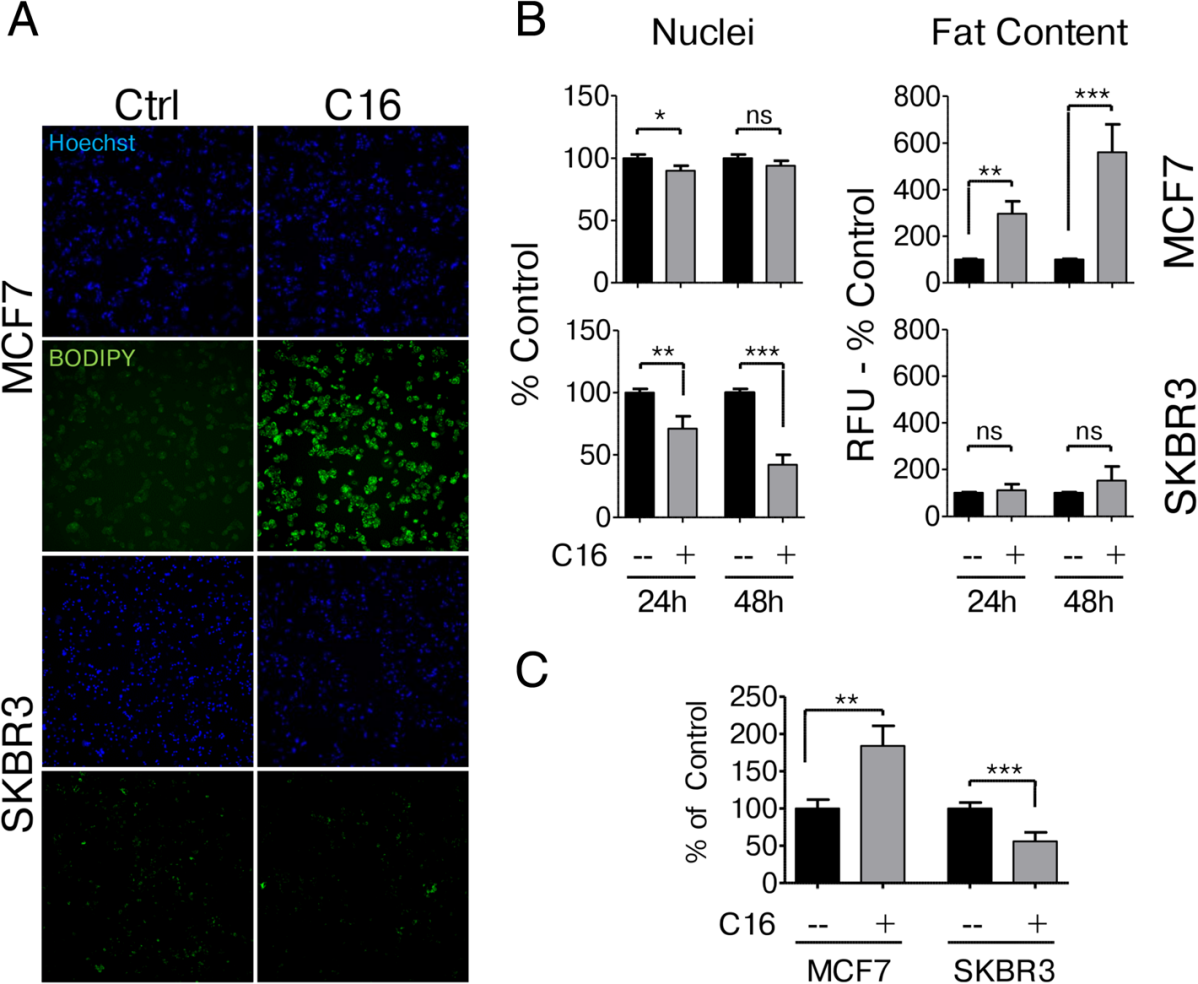
HER2/neu-positive SKBR3 and HER2-normal MCF7 cells differ in their response to palmitate. **a**, **b** HER2/neu-positive SKBR3 and HER2-normal MCF7 breast cancer cells were treated with 250 μM palmitate or vehicle for 24 h and 48 h. Cells were fixed and neutral lipids were stained with BODIPY 493/503. Nuclei were stained with Hoechst 33342. BODIPY fluorescence and nuclei were imaged and quantified using the INCell Analyzer 2200 and INCell Investigator software. C16 = palmitate. **c** Exogenous palmitate alters anchorage-independent growth. Cells were plated in ultra-low attachment plates in the presence of 250 μM palmitate or vehicle control and incubated for 11 days. Viable cells were quantified using the Alamar Blue cell health indicator. Statistical analysis was carried out in Graphpad Prism. Data are presented as mean ± SD. * = *p* · 0.05, ** = *p* · 0.01, *** = *p* · 0.001, Student’s t-test, *n* = 3, *n* = 4 in C

The HER2-normal cell line MCF7 and the HER2/neu-positive SKBR3 cell line were used to investigate the differential effects of low concentrations of exogenous palmitate. The use of MCF7 cells as a control is preferable since they can be grown in the same cell culture medium as SKBR3 cells and previous studies have shown that the response to exogenous palmitate in MCF7 cells is comparable to that of non-tumorigenic MCF10A mammary epithelial cells or normal human mammary epithelial cells (HMECs) [7, 26]. We cultured MCF7 and SKBR3 cells in the presence of either 250 μM palmitate or vehicle control and monitored cell count as well as the levels of intracellular neutral fat stores. We chose this concentration based on previous studies showing that fasting FFA concentrations in plasma/serum are in the range of 300–600 μM [12–14] with palmitate representing about one quarter of the total FFAs [15, 16]. Exogenous palmitate rapidly reduces the number of SKBR3 cells, but not MCF7 cells. These effects are mediated by the effects of palmitate on cellular physiology and not as effects on cellular integrity which are not seen at concentrations in this range (Additional file 1: Figure S1). While SKBR3 cells show higher basal levels of stored neutral fats that do not change with palmitate treatment, MCF7 cells display low basal neutral fat content which increases significantly upon palmitate exposure (Fig. 1a, b). Under anchorage-independent growth conditions exogenous palmitate appears to be beneficial to the growth of MCF7 cells but detrimental to that of SKBR3 cells (Fig. 1c).

**Fig. 2:**
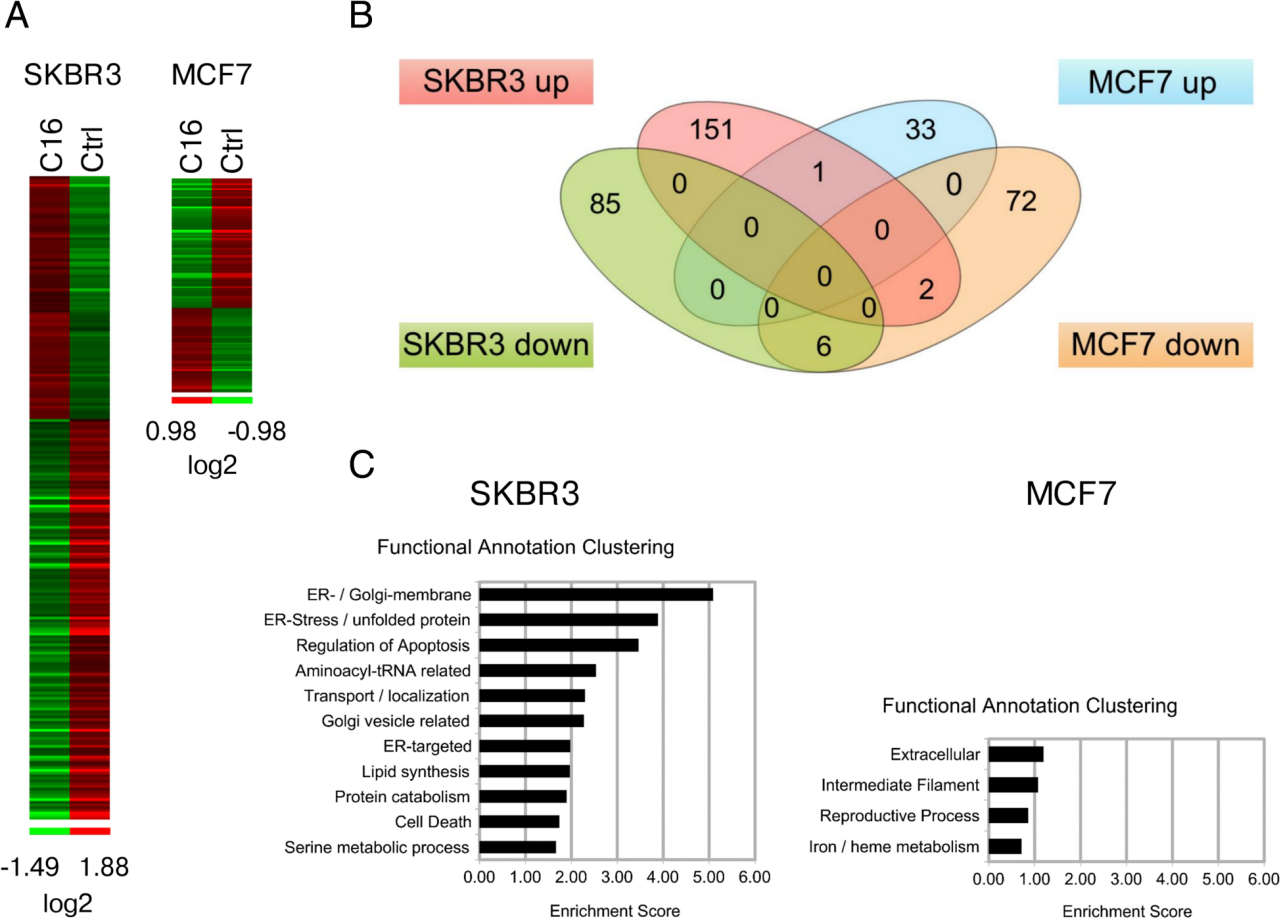
Exogenous palmitate induces a distinct transcriptional response in HER2/neu-positive SKBR3 breast cancer cells. HER2/neu-positive SKBR3 and HER2-normal MCF7 breast cancer cells were treated with 250 μM palmitate or vehicle for 24 h. C16 = palmitate. Total RNA was harvested and subjected to microarray analysis using Affymetrix Gene ST 1.0 arrays. **a** Heatmap showing log2-ratios of altered transcripts between vehicle and palmitate treated samples (1.5-fold cut-off). **b** Venn diagram showing the number of genes that are up- or downregulated with palmitate treatment in SKBR3 and MCF7 cells (1.5-fold cut-off). **c** Results of the Functional Annotation Clustering analysis after GO-term enrichment of upregulated genes using the DAVID Bioinformatics Resource

To identify transcriptional programs and signaling networks that mediate the response to exogenous palmitate in HER2/neu-positive and HER2-normal breast cancer cells we performed microarray analysis. SKBR3 and MCF7 cells were treated with 250 μM palmitate or vehicle control for 24 h, total RNA was isolated and hybridized to Affymetrix Gene ST 1.0 arrays. Figure 2a shows a heatmap representation of the transcripts that display at least 1.5-fold change in expression. In MCF7 cells, 34 genes are upregulated and 80 genes are downregulated in response to palmitate treatment. Whereas in SKBR3 cells, 154 genes show increased expression and 91 genes show decreased expression. Additionally, the extent of the transcriptional changes is more pronounced in the SKBR3 cells compared to MCF7 cells. The transcripts that are modulated by exogenous palmitate are also remarkably different between the two cell types (Fig. 2b), with only one gene being jointly upregulated and six shared genes in the downregulated group.

We performed gene ontology (GO) enrichment analysis using the DAVID Bioinformatics Resource to investigate which transcriptional programs are regulated in the two cell lines in response to exogenous palmitate [20, 21]. Figure 2c shows the results of a functional annotation clustering analysis which combines similar GO-terms into clusters. The enrichment score for each cluster indicates the significance compared to the whole dataset. Upregulated GO-terms in MCF7 cells show little, if any, functional enrichment, with the most enriched GO-terms being related to “extracellular” (ES: 1.19) and “intermediate filament” (ES: 1.08). Upregulated GO-terms in SKBR3 cells however cluster around terms related to “ER-Golgi-membrane (ES: 5.09), ”ER stress/unfolded protein (ES: 3.89) and “regulation of apoptosis” (ES: 3.47), indicating the induction of ER stress as well as apoptotic signaling.

A detailed list of the enriched GO-terms for up-and downregulated genes in both cell lines can be found in the supplementary material (Additional file 1: Figure S2 and Additional file 1: Figure S3). Of note, in SKBR3 cells, exogenous palmitate significantly attenuates processes related to DNA replication, cell cycle and cell division (Additional file 1: Figure S3). We used the Cytoscape plugin Enrichment Map to better visualize the functional relationships between the significantly enriched GO-terms in SKBR3 cells, as well as illustrate gene member overlap (Additional file 1: Figure S4). The top clusters “ER stress/UPR”, “apoptosis” and “cell cycle” are immediately apparent from the map. To further elucidate the transcriptional response to exogenous palmitate in SKBR3 cells we performed gene set enrichment analysis (GSEA) [27, 28]. Genes that show increased expression after palmitate treatment in SKBR3 cells overlap significantly with genes that are upregulated after proteasome inhibition by epoxomicin, supporting the claim of an ER stress/UPR response in the SKBR3 cells (Additional file 1: Figure S5). However, palmitate does not directly affect proteasome activity in SKBR3 cells (Additional file 1: Figure S6). Genes that are downregulated in SKBR3 cells after palmitate treatment overlap significantly with a group of genes that show decreased expression after growth factor deprivation (Additional file 1: Figure S5).

### Palmitate induces G2-phase cell cycle delay and apoptosis in HER2/neu-positive SKBR3 breast cancer cells

**Fig. 3:**
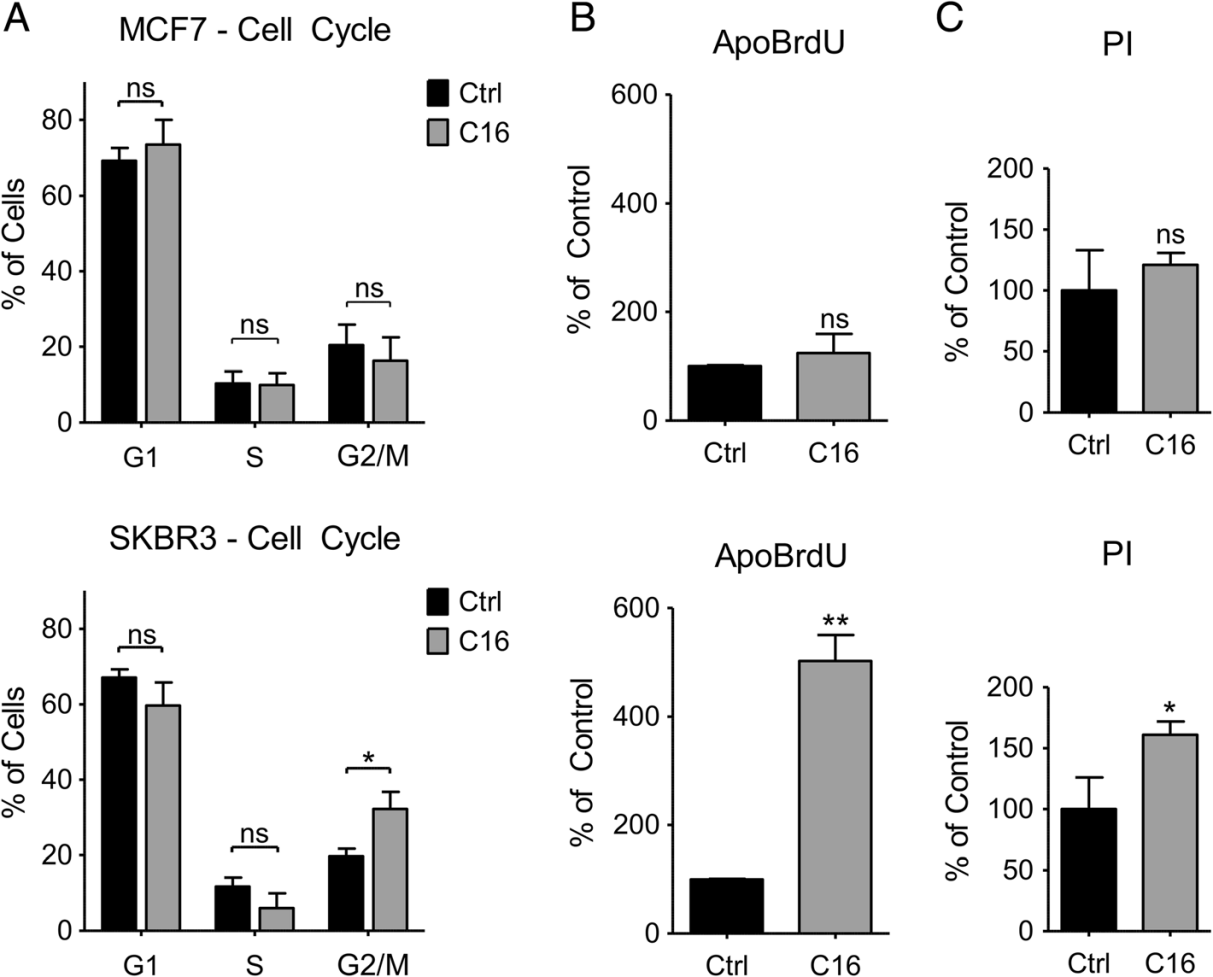
Palmitate induces G2-phase cell cycle delay and apoptosis in HER2/neu-positive SKBR3 breast cancer cells. **a** MCF7 and SKBR3 cells were treated with 250 μM palmitate or vehicle for 24 h. C16 = palmitate. Cells were ethanol-fixed and DNA content was analyzed by propidium iodide staining and flow cytometry analysis. Cell cycle phases were determined using FlowJo software. **b** MCF7 and SKBR3 cells were treated with 250 μM palmitate or vehicle for 48 h, fixed in paraformaldehyde and subjected to Apo-BrdU DNA end-labeling. A FITC-labeled anti-BrdU antibody was used to detect incorporated BrdU by flow cytometry. **c** MCF7 and SKBR3 cells were treated with 250 μM palmitate or vehicle for 24 h. Dead cells were monitored for propidium iodide uptake via flow cytometry. Data were analyzed using FlowJo software and normalized to control conditions. Statistical analysis was carried out in Graphpad Prism. Data are presented as mean ± SD. * = *p* · 0.05, ** = *p* · 0.01, Student’s t-test, *n* = 3

The computational analysis suggests that exogenous palmitate induces changes in cell cycle regulation as well as apoptotic signaling, so we performed cell cycle analysis by flow cytometry. As expected, the HER2-normal MCF7 breast cancer cells show no changes in the cell cycle phase distribution, comparing palmitate-treated and vehicle-treated cells. HER2/neu-positive SKBR3 cells however show a significant delay in the G2 phase of the cell cycle when treated with exogenous palmitate (Fig. 3a). Induction of apoptosis was assessed by terminal deoxynucleotidyl transferase-mediated DNA-end labeling with ApoBrdU, whereas cell death was monitored by propidium iodide (PI) uptake. Exogenous palmitate causes a significant increase in DNA fragmentation in HER2/neu-positive SKBR3 cells, but has no effect on HER2/neu-normal MCF7 cells (Fig. 3b). The PI-positive SKBR3 cell population increases significantly as well (Fig. 3c). This indicates that exogenous palmitate induces apoptosis and cell death in HER2/neu-positive SKBR3 cells but not HER2/neu-normal MCF7 cells, confirming the predictions made by the functional enrichment analyses.

### Logistic Regression analysis identifies transcription factor binding motif enrichment for XBP1 and ATF6

**Fig. 4:**
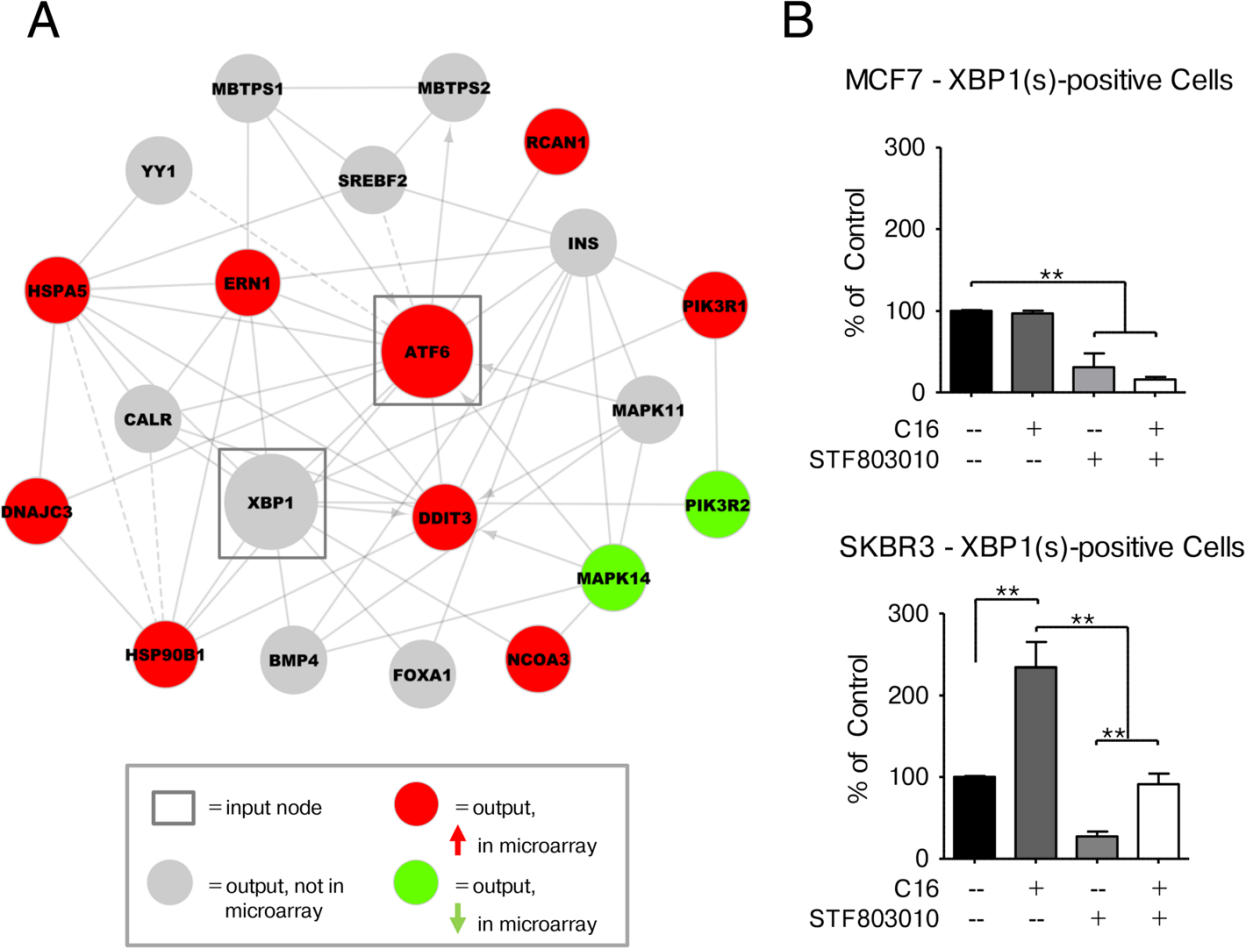
Logistic Regression analysis determines transcription factor binding motif enrichment for XBP1 and ATF6. **a** Interaction network adapted from the STRING database showing the immediate network neighborhood of the two enriched transcription factors ATF6 and XBP1 obtained through the analysis described in Table 1. Big nodes indicate input genes, red nodes indicate increased expression, green nodes indicate decreased expression as identified in the microarray analysis described in Fig. 2. Grey nodes are part of the interaction network but were not identified in the microarray results. **b** MCF7 and SKBR3 cells were transfected with the transcriptional reporter construct pCAX-XBP1-ΔDBD-venus which results in the expression of a functional fluorescent XBP1-venus fusion protein after mRNA splicing by the ER stress regulator IRE1. STF803010 inhibits the RNase activity of IRE1 (+ = 10 μM). The cells were treated with 250 μM palmitate 24 h post-transfection, alone or in combination with STF803010 and green fluorescent cells were counted using the InCell Analyzer 2200. C16 = palmitate. The data were normalized to control conditions and are presented as mean ± SD. ** = *p* · 0.01, One-way ANOVA with Bonferroni post-test. *n* = 3

To further elucidate which transcriptional programs are activated by palmitate in the HER2/neu-positive SKBR3 breast cancer cell line, we performed logistic regression analysis using the LRPath web resource [22]. By querying the TRANSFAC database, the algorithm can identify significantly enriched transcription factor binding motifs in the up- and downregulated gene groups. We identified the transcription factors ATF6 (FDR q-value: 2.69 × 10-3) and XBP1 (FDR q-value: 3.43 × 10-3) as the two most significantly enriched motifs in the upregulated genes. The two most significantly enriched transcription factor binding motifs in the downregulated genes are currently not known to be bound by any particular transcription factor (Table 1). ATF6 and XBP1 are both known regulators of the ER stress and unfolded protein response (UPR) [29]. The two transcription factors were used as input for the STRING database of protein interactions to retrieve the immediate network neighborhood [24], which is shown as a Cytoscape network in Fig. 4a. ATF6 expression increases significantly in palmitate-treated SKBR3 cells, however, no changes in XBP1 mRNA levels were detected (Fig. 2 and Fig. 4).We used a transcriptional reporter construct described by Samali et al. to evaluate whether exogenous palmitate is able to induce XBP1 splicing, which is associated with the activation of ER stress [25]. MCF7 and SKBR3 cells were transfected with the pCAX-XBP1-ΔDBD-venus vector which results in the expression of a functional fluorescent XBP1-venus fusion protein if the XBP1 mRNA is spliced by the ER stress activator, IRE1. The DNA-binding domain has been deleted from the reporter construct to prevent the fusion-protein from interfering with the function of the endogenous XBP1(s) protein. Twenty-four hours post-transfection, the cells were challenged with exogenous palmitate for 24 h either alone or in the presence of the IRE1 inhibitor STF803010 and fluorescent cells were counted. While both cell lines show low levels of XBP1 splicing in control conditions, only the HER2/neu-positive SKBR3 cells exhibit a significant increase in IRE1-dependent XBP1 splicing after palmitate treatment (Fig. 4b). These data indicate that exogenous palmitate induces IRE1-mediated XBP1 splicing in the HER2/neu-positive SKBR3 cells, but not the HER2/neu-normal MCF7 cells, confirming, in part, the computational predictions.

**Table 1:** Results of the logistic regression analysis using LRPath

Gene-set ID	# of genes	Hits	*p*-Value	FDR	Regulation	Transcription factor
ATF6_01	93	34	2.23 × 10^-5^	2.69 × 10^-3^	up	ATF6
XBP1_01	152	58	4.27 × 10^-5^	3.43 × 10^-3^	up	XBP1
E2F_02	96	96	1.05 × 10^-14^	2.54 × 10^-12^	down	N/A
NFY Q6	26	16	1.06 × 10^-3^	3.65 × 10^-2^	down	N/A

A ranked list consisting of up- and downregulated genes in HER2/neu-positive SKBR3 cells after palmitate treatment was used as input for a logistic regression pathway analysis (LRPath) to determine enriched transcription factor binding motifs based on the TRANSFAC database. The table shows the two most significantly enriched motifs in the upregulated and downregulated gene groups, as well as the associated transcription factors. N/A indicates that no known transcription factor is associated with this particular motif

### Exogenous palmitate increases DDIT3/CHOP expression in HER2/neu-positive SKBR3 cells

**Fig. 5:**
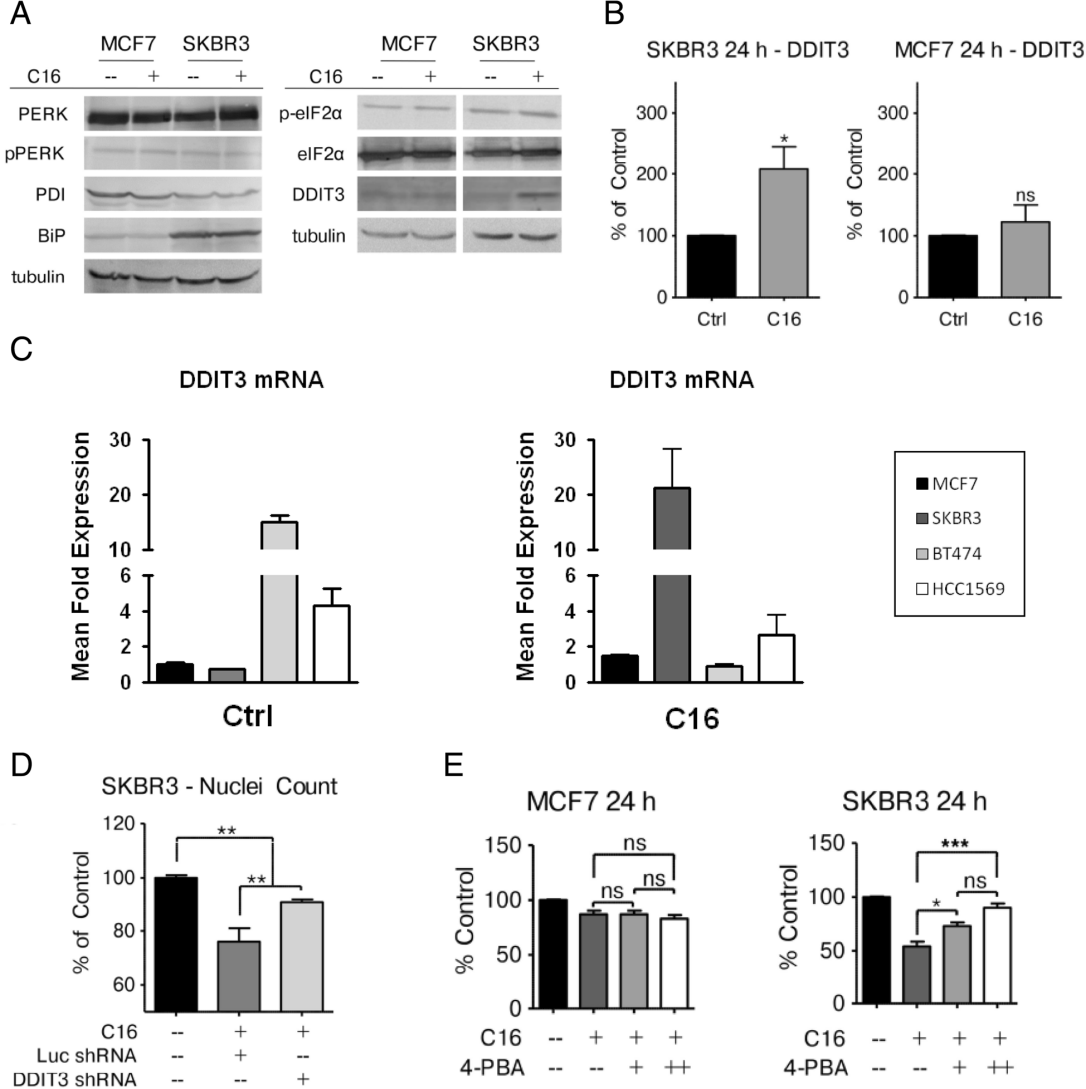
Exogenous palmitate increases DDIT3/CHOP expression in HER2/neu positive breast cancer cells. **a** MCF7 and SKBR3 cells were treated with 250 μM palmitate or vehicle control for 24 h. C16 = palmitate. Total protein was harvested and subjected to western blot analysis to evaluate the protein expression changes for the ER stress related markers PERK, phospho-PERK (pPERK, Thr980), PDI, BiP, eIF2α, phospho-eIF2α (p-eiF2α, Ser51) and DDIT3/CHOP. Each experiment was performed in triplicate, representative images are shown. **b** Quantification of the DDIT3/CHOP western blots using ImageJ. **c** Cells were treated with vehicle for 24 h and assessed for basal levels of DDIT3, normalized to MCF7 levels. Quantification of DDIT3 mRNA levels after treatment with 500 μM sodium palmitate for 24 h. The data are presented as mean ± SEM of three independent experiments. **d** SKBR3 cells were transfected with an shRNA targeting DDIT3/CHOP or luciferase, which has no target in these cells. 48 h post-transfection the cells were treated with 250 μM palmitate or vehicle for 24 h and nuclei were counted using the INCell Analyzer 2200. **e** MCF7 and SKBR3 cells were treated with 250 μM palmitate or vehicle either alone or in the presence of the chemical chaperone 4-PBA for 24 h. Nuclei were counted and the data were normalized to control conditions. Data are presented as mean ≤ SD. * = *p* ≤ 0.05, ** = *p* ≤ 0.01, *** = *p* ≤ 0.001. One-way ANOVA with Bonferroni post-test. *n* = 3

The activation of IRE1 and ATF6, two of the three major regulators of ER stress, and the computational predictions suggest that the activation of a canonical ER stress response occurs in SKBR3 cells exposed to exogenous palmitate. We performed western blot analyses for other proteins that are known to be involved in the ER stress and unfolded protein response pathways to further evaluate these predictions (Fig. 5a). Interestingly, we did not detect any changes in the protein levels or relative phosphorylation levels for the third major regulator of ER stress, PKR-like endoplasmic reticulum kinase (PERK). The primary substrate of PERK is the eukaryotic translation initiation factor 2 alpha (eIF2α), a critical regulator of translation and the unfolded protein response. Upon activation, PERK phosphorylates eIF2α, which attenuates global protein synthesis and leads to the preferred translation of stress response-related transcripts. In accordance with the results for PERK activation, palmitate treatment does not induce any changes in eIF2α protein expression or relative phosphorylation levels in either MCF7 or SKBR3 cells. Protein disulfide isomerase (PDI) and GRP78/BiP, both known to be involved in the unfolded protein response, are not increased at the protein level with palmitate treatment in either cell line. There is, however, a substantial increase in the expression of the pro-apoptotic regulator of ER stress and the unfolded protein response, DDIT3/CHOP, in SKBR3 cells. Treatment with exogenous palmitate increases DDIT3/CHOP protein levels in the HER2/neu-positive SKBR3 cells by approximately 2-fold, whereas HER2/neu-normal MCF7 cells are not affected (Fig. 5b). Analysis of MCF7 and SKBR3 cells showed that they have similar basal levels of DDIT3/CHOP. Notably, other HER2/neu-positive breast cancer cell lines, BT474 and HCC1569, have elevated DDIT3/CHOP mRNA levels under standard growth growth conditions. Treatment with palmitate increased DDIT3/CHOP mRNA levels greater than 20-fold in SKBR3 cells with a lesser induction seen in HCC1569 and BT474, most likely due to their high basal levels (Fig. 5c). A similar situation existed with levels of ATF6 and spliced XBP1 in the HCC1569 and BT474 cells which were significantly increased compared to MCF7 cells but exhibited relatively modest levels of induction due to palmitate (Additional file 1: Figure S7).

Since DDIT3/CHOP is known to induce pro-apoptotic signaling we investigated whether the increase in DDIT3/CHOP expression with palmitate treatment is responsible for the increase in cell death in the SKBR3 cell line. Cells were transfected with an shRNA construct targeting DDIT3 or luciferase as a control and were subsequently treated with palmitate or vehicle control (Fig. 5d). Knockdown of DDIT3/CHOP significantly reduces the cytotoxic effects of exogenous palmitate in SKBR3 cells, compared to the palmitate-treated and control-transfected conditions. However, the protection conferred by DDIT3/CHOP knockdown is not complete as there is a significant difference between the palmitate-treated DDIT3/CHOP -knockdown cells and vehicle-treated cells, indicating that other factors besides DDIT3/CHOP might contribute to the toxic effects of exogenous palmitate in HER2/neu-positive SKBR3 breast cancer cells. These data corroborate the induction of ER stress/UPR in SKBR3 cells treated with exogenous palmitate, as predicted by our computational analyses. Additionally, despite the genetic heterogeneity of HER2/neu-positive breast cancer [1], aspects of this response may occur generally in HER2/neu-positive breast cancer cell lines which our previous work has shown undergo apoptosis when cultured with exogenous palmitate [6, 7]. To investigate whether unfolded or misfolded proteins contribute to the toxic effects of palmitate in these cells we used the chemical chaperone 4-phenylbutyric acid (4-PBA), which is able to increase the protein folding capacity of the ER and attenuate the unfolded protein response [30]. SKBR3 cells display a dose dependent attenuation of palmitate-induced cytotoxicity when 4-PBA is co-administered with palmitate, whereas MCF7 cells show no change related to the addition of 4-PBA (Fig. 5e).

To determine whether ER stress induction might occur in tumors, we analyzed molecular profiling data sets of invasive breast carcinomas generated by the TCGA Research Network: http://cancergenome.nih.gov/ [1]. Analysis of these data reveal an association between ER stress regulators and the regulators of the lipogenic phenotype previously described in HER2/neu-positive breast cancer cell lines [6, 7]. Statistically significant greater than twofold increases in expression in ER stress pathway genes are found to co-occur with increased levels of HER2/neu, NR1D1 and PBP in human breast tumors (Additional file 1: Figure S8). In this data set, approximately 15 % of the tumors exhibit increased expression of HER2/neu. 43 % of tumors that exhibit increased expression of PERK and 39 % of tumors that exhibit increased expression of IRE1 are found in the HER2/neu increased expression group. 53 % of the tumors that overexpress HER2/neu, NR1D1 and PBP together also overexpress one or more ER stress marker. The importance of these statistically significant associations is difficult to interpret unequivocally since the increased expression of each ER stress marker seems to be somewhat independent and since the cellular composition of tumors is generally heterogeneous. However, it lends support to the notion that the frequent increase in lipogenesis that occurs in HER2/neu-positive breast cancers may trigger some degree of ER stress response in vivo.

### Palmitate decreases HER2 and HER3 expression and sensitizes the cells to trastuzumab treatment

**Fig. 6:**
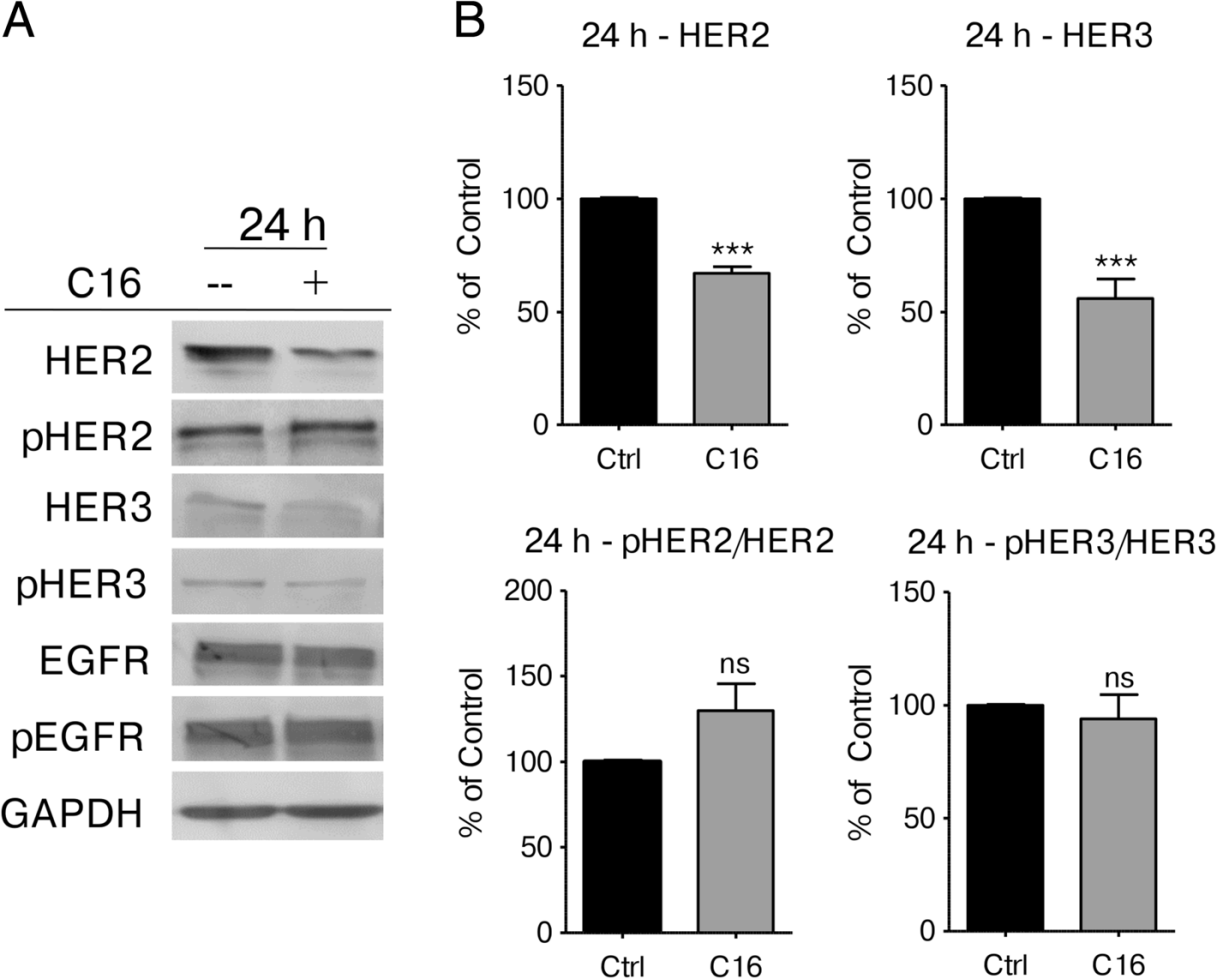
Exogenous Palmitate decreases HER2 and HER3 expression in HER2/neu-positive SKBR3 cells. **a** SKBR3 cells were treated with 250 μM palmitate or vehicle for 24 h. C16 = palmitate. Total protein was harvested and subjected to western blot analysis to evaluate the protein expression changes for the HER-family proteins HER2, phospho-HER2 (p-HER2, Tyr1221/1222), HER3, phospho-HER3 (pHER3, Tyr1289)), EGFR and phospho-EGFR (pEGFR,Tyr1068). Each experiment was performed in triplicate, representative images are shown. **b** Quantification of the HER2, pHER2, HER3 and pHER3 western blots using ImageJ. **c** Intensities were normalized to loading control and vehicle-control. The data is presented as mean · SD. * = *p* · 0.05, ** = *p* · 0.01, *** = *p* · 0.001. Student’s t-test. *n* = 3

**Fig. 7:**
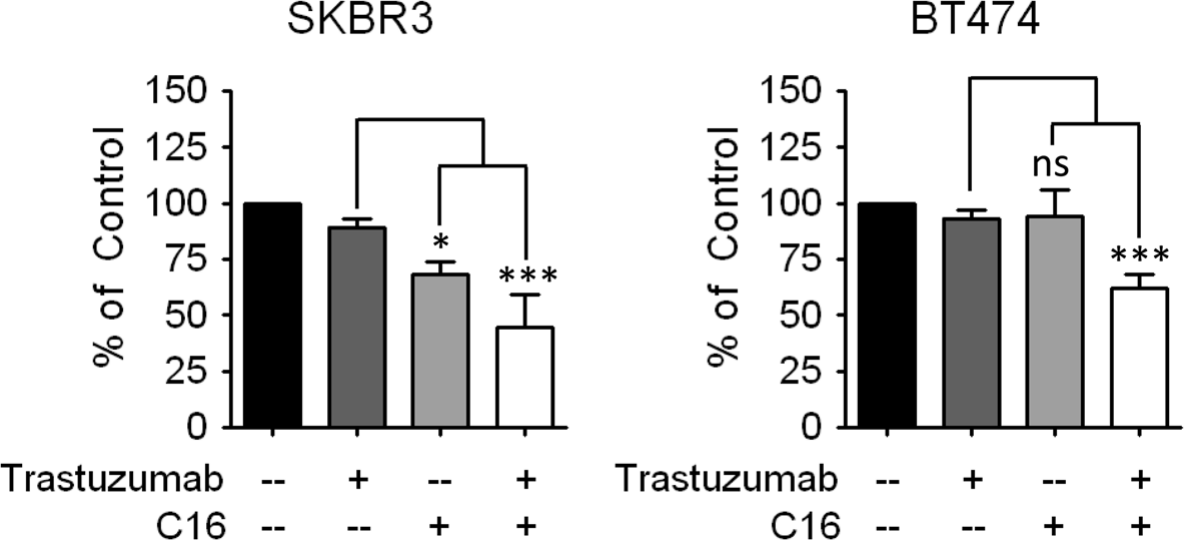
Exogenous palmitate sensitizes HER2/neu-positive cells to trastuzumab. The HER2/neu-positive breast cancer cell lines SKBR3 and BT474 were treated with 22 μg/mL trastuzumab for 48 h either with palmitate (+ = 150 μM, ++ = 250 μM) or vehicle pre-treatment. C16 = palmitate. Nuclei were counted using the INCell Analyzer 2200. The data were normalized to 48 h trastuzumab alone and are presented as mean · SD. * = *p* · 0.05, ** = *p* · 0.01, *** = *p* · 0.001. One-way ANOVA with Bonferroni post-test. *n* = 3

Based on the GSEA analysis, genes that display decreased expression after palmitate treatment in HER2/neu-positive SKBR3 cells significantly overlap with genes that are downregulated after growth factor deprivation (Additional file 1: Figure S5). A probable cause for these transcriptional changes in HER2/neu-positive cells is the deregulation of HER2 signaling after palmitate treatment. To test this hypothesis, we evaluated protein expression changes in the HER-family of receptor tyrosine kinases in palmitate-treated SKBR3 cells by western blot. Exogenous palmitate induces a significant reduction in the HER2 and HER3 protein levels, while EGFR expression (HER1) and phosphorylation is unaffected. Interestingly, the relative phosphorylation levels of HER3 remain unchanged as well, indicating a reduction in total HER3 as well as active, phosphorylated HER3 (Fig. 6a, b). The relative phosphorylation of HER2 may be increased by palmitate treatment; however the differences are not significant. Previous studies have shown that HER2 and HER3 heterodimerize and form the predominant and most potent mitogenic combination in carcinoma cells [31–33], hence we investigated whether these particular effects of exogenous palmitate on HER2/neu-positive SKBR3 breast cancer cells are able to increase the efficacy of trastuzumab treatment. SKBR3 cells were pretreated with different doses of palmitate (150 μM and 250 μM) or vehicle control for 24 h. The palmitate-containing medium was removed and replaced with fresh medium containing 22 μg/mL trastuzumab [19] or vehicle control and the cells were incubated for an additional 48 h. Palmitate significantly increases the growth inhibitory effects of trastuzumab compared to vehicle control (Fig. 7). This effect is also found to occur in the HER2/neu-positive breast cancer cell line, BT474. The somewhat attenuated effect in BT474 cells is congruent with our observations that BT474 cells are less sensitive to exogenous palmitate than SKBR3 cells. In either case, the growth inhibitory and ER stress inducing effects of palmitate on HER2/neu-positive breast cancer cells are not caused by the decrease in HER2 and HER3 levels. Overexpression of HER2/neu in a normal breast line does not by itself counteract the effects of palmitate on reducing cell numbers (Additional file 1: Figure S9A). Similarly, the induction of ER stress markers after palmitate treatment is largely unaffected by HER2/neu overexpression (Additional file 1: Figure S9B). Together, these results provide additional evidence for a link between fatty acid synthesis and HER2/neu function to what is already known [6, 10, 34, 35]. Additionally, they are consistent with previous work showing that the lipogenic phenotype of HER2/neu-positive breast cancer cells is dependent on genes other than HER2/neu itself [6, 7]. It will undoubtedly take additional experimentation to unravel the signaling interactions related to the effects of palmitate on HER2 and HER3 levels.

## Discussion

Our previous work has shown that HER2/neu-positive breast cancer cells contain high levels of endogenous saturated fatty acids and neutral lipids and generally exhibit a pro-lipogenic phenotype [6–9, 19]. This Warburg-like physiology relies on active fatty acid synthesis for survival and aggressive behavior. Since small amounts of exogenously supplied palmitate are toxic to HER2/neu-positive breast cancer cells, however, this phenotype might represent an Achilles heel [7]. In this study, we used physiological concentrations of exogenous palmitate both to investigate molecular mechanisms of lipotoxicity associated with this Warburg-like physiology and to model high levels of dietary saturated fat. We identified pathways that are modulated by exogenous palmitate in the HER2/neu-positive SKBR3 breast cancer cell line and compared this response to that of HER2-normal MCF7 breast cancer cells, which have previously been shown to respond to exogenous palmitate in a way that is comparable to non-tumorigenic MCF10A mammary epithelial cells or normal human mammary epithelial cells (HMECs) [7, 26]. This analysis was extended to additional HER2/neu-positive breast cancer cell lines and publicly available molecular profiling data of human breast tumors.

Exogenous palmitate induces distinct transcriptional responses in SKBR3 and MCF7 cells, which are congruent with the severity of the observed toxicity (Fig. 2 and [6]). Most notably in HER2/neu-positive SKBR3 cells, exogenous palmitate induces a partial ER stress response through the activation of the IRE1-XBP1 and ATF6 axes, but not the PERK-eIF2α axis, which ultimately leads to CHOP-dependent cell death (Fig. 5). Increased levels of ER stress response markers are also found in HER2/neu-positive breast cancer cell lines and human breast tumors. The general trend of the three HER2/neu-positive breast cancer cell lines tested was that they possessed higher basal levels of DDIT3/CHOP, ATF6 and spliced XBP1 and that these levels increased in response to palmitate treatment (Additional file 1: Figure S7). These effects were not observed in the other breast cells tested. Additionally, analysis of molecular profiling data sets of invasive breast carcinomas generated by the TCGA Research Network [1] revealed an association between the ER stress markers and the HER2/neu, NR1D1 and PBP genes linked to lipogenesis [6–9, 19] in human breast tumors (Additional file 1: Figure S8). Together, these data point to ER stress as a potential consequence of increased palmitate levels in HER2/neu-positive breast cancer cells, although the precise pathway may not be clear at present. Several studies have reported that palmitate induces ER stress responses in liver and pancreatic beta-cells. Cao et al. report that palmitate induces ER stress and apoptosis in liver cells through the activation of PERK/ATF4/CHOP [36]. Sommerweis et al. obtained similar results in pancreatic beta-cells, where palmitate induced PERK activation and eIF2α phosphorylation but no upregulation of ATF6 was detected [37]. These studies have identified palmitate induced ER stress pathways that are somewhat different from what we have found in the HER2/neu-positive breast cancer cells. However, the overexpression of HER2, which has been linked to increased sensitivity towards ER stress-inducing agents [38], may complicate the interpretation. Similar to our results, Young et al. report saturated fatty acid-induced ER stress and CHOP-dependent cell death in mouse embryonic fibroblasts (MEFs) with consitutively active mTOR signaling when the cells are cultured under hypoxic conditions [39]. mTOR signaling is frequently activated in HER2/neu-positive breast cancer cells and tumors and may contribute to the lipotoxicity observed in HER2/neu-positive SKBR3 cells (reviewed in [9]).

Determination of the molecular mechanism of palmitate in inducing ER stress and reducing HER2/HER3 levels will require additional study. Previous studies have suggested that exogenous palmitate exerts its toxic effects through the dysregulation of protein palmitoylation. Baldwin et al. found that palmitate-induced ER stress and apoptosis in beta-cells could be ameliorated by adding the palmitoylation inhibitor 2-bromopalmitate [40] and the ER-resident chaperone, calnexin, has been shown to be functionally regulated by palmitoylation. Upon ER stress induction, palmitoylation of calnexin decreases, which promotes its chaperone function [41]. However, this does not appear to be a relevant mechanism in HER2/neu-positive breast cancer cells. 2-bromopalmitate appears to be even more toxic to these cells than palmitate (Baumann et al., unpublished observations). These results are consistent with a metabolic effect as 2-bromopalmitate inhibits a variety of enzymes, some of which are required for TAG formation, a process that has been shown to be critical for the survival of HER2/neu-positive breast cancer cells [6, 7, 42].

Given the altered metabolic phenotype of HER2/neu-positive breast cancer cells, which rely on active fatty acid synthesis for survival [6, 7], it is possible that exogenous palmitate induces ER stress and decreases HER2/HER3 protein levels indirectly by interfering with FA synthesis. Upon uptake into the cell, palmitate is converted into palmitoyl-CoA which is a major allosteric feedback inhibitor of ACACA, the rate-limiting enzyme in fatty acid synthesis [43]. The inhibition of fatty acid synthesis caused by palmitate (Baumann et al. unpublished) may have effects on growth factor signal transduction. Several lines of investigation have linked HER2 and fatty acid synthase (FASN) previously. ACACA and FASN are upregulated in HER2/neu-positive breast cancers at the transcriptional [6] and the translational level [10]. Overexpression of FASN in immortalized, non-tumorigenic mammary epithelial cells has been shown to induce HER2 overexpression and activation [34] and there are reports that FASN may be directly phosphorylated by HER2 [35]. Additionally, pharmacological inhibition of FASN induces ER stress [44], decreases HER2 protein levels [45, 46], sensitizes cells to trastuzumab treatment [46] and reverses acquired autoresistance to trastuzumab [47, 48]. All of these findings are consistent with our observations that exogenous palmitate induces ER stress, decreases HER2 and HER3 protein levels and sensitizes the cells to trastuzumab-mediated growth inhibition. These data suggest that exogenous palmitate may function, at least in part, as a feedback inhibitor of fatty acid synthesis in HER2/neu-positive breast cancer cells, as well as an ER stress inducer. Since prolonged activation of the ER stress response has previously been linked to increased sensitivity to various chemotherapeutic agents, including trastuzumab [49] as we see (Fig. 7), levels of palmitate in the breast cancer microenvironment may have positive impacts on treatment. Along these lines, high levels of dietary saturated fatty acids may be capable of interfering with HER2 expression and signaling during disease development. While many studies have shown a protective effect of polyunsaturated fatty acids on breast cancer development in general, elevated levels of saturated fat may impose a metabolic constraint that specifically decreases the development of HER2/neu-positive breast cancer - a situation consistent with recent epidemiological studies [11]. Although consumption of high levels of saturated fat is not an envisioned therapy, further investigation of this phenomenon may lead to an improved understanding of breast cancer cell physiology. In any event, this study provides further evidence that HER2 signaling, fatty acid metabolism and ER stress signaling are highly integrated processes that may be important for disease development and progression.

## Conclusions

We have shown that physiological concentrations of exogenous palmitate induce a partial ER stress response and CHOP-dependent apoptosis in HER2/neu-positive breast cancer cells. This ER stress response is accompanied by a significant reduction in HER2 and HER3 protein levels and sensitizes cells to trastuzumab treatment. These data provide further evidence that HER2 signaling and fatty acid metabolism are highly integrated processes that may be important for disease development and progression.

## Abbreviations

4-PBA, 4-phenylbutyric acid; ACACA, acetyl-CoA carboxylase alpha; ACLY, ATP-citrate lyase; C16, palmitate; Ctrl, control; DPBS, Dulbecco’s phosphate buffered saline; ES, enrichment score; FA, fatty acid; FASN, fatty acid synthase; FBS, fetal bovine serum; GSEA, gene set enrichment analysis; HMECs, human mammary epithelial cells; HRP, horseradisch peroxidase; luc, luciferase; PBP, PPARγ binding protein; PERK, PKR-like endoplasmic reticulum kinase; RIN, RNA integrity number; SDS-PAGE, sodium dodecylsulfate polyacrylamide gel electrophoresis; TAG, triacylglycerides; TBS, Tris buffered saline; UPR, unfolded protein response

## Additional file

Additional file 1: Figure S1. Addition of exogenous palmitate does not increase cellular permeability of SKBR3 cells. Figure S2: Detailed results of the GO-term enrichment analysis for MCF7 cells using the DAVID Bioinformatics resource. Figure S3: Detailed results of the GO-term enrichment analysis for SKBR3 cells using the DAVID Bioinformatics resource. Figure S4: Enrichment map illustrating gene member overlap in the enriched GO-terms for SKBR3 cells treated with palmitate. Figure S5: Gene set enrichment analysis of significantly altered transcripts in SKBR3 cells after palmitate treatment. Figure S6: Exogenous palmitate does not alter the proteasome activity in HER2/neu-positive SKBR3 breast cancer cells. Figure S7: Expression of ER stress markers in HER2/neu-positive breast cancer cells upon treatment with palmitate. Figure S8: Alterations of ER stress regulators associate with alterations of regulators of the lipogenic phenotype previously described in HER2/neu-positive breast cancer cell lines. Figure S9: Her2/neu expression by itself has relatively minor effects on ER stress. Table S1: Primers used in expression analysis of ER stress markers. (DOCX 2241 kb)
