# Supplementary material for: Palmitate-induced ER stress increases trastuzumab sensitivity in HER2/neu-positive breast cancer cells
Source: BMC Cancer. 2016 Jul 27;16:551. doi: 10.1186/s12885-016-2611-8 (PMC4964104; doi:10.1186/s12885-016-2611-8)
Supplement: Additional file 1: Figure S1. — Addition of exogenous palmitate does not increase cellular permeability of SKBR3 cells. Figure S2: Detailed results of the GO-term enrichment analysis for MCF7 cells using the DAVID Bioinformatics resource. Figure S3: Detailed results of the GO-term enrichment analysis for SKBR3 cells using the DAVID Bioinformatics resource. Figure S4: Enrichment map illustrating gene member overlap in the enriched GO-terms for SKBR3 cells treated with palmitate. Figure S5: Gene set enrichment analysis of significantly altered transcripts in SKBR3 cells after palmitate treatment. Figure S6: Exogenous palmitate does not alter the proteasome activity in HER2/neu-positive SKBR3 breast cancer cells. Figure S7: Expression of ER stress markers in HER2/neu-positive breast cancer cells upon treatment with palmitate. Figure S8: Alterations of ER stress regulators associate with alterations of regulators of the lipogenic phenotype previously described in HER2/neu-positive breast cancer cell lines. Figure S9: Her2/neu expression by itself has relatively minor effects on ER stress. Table S1: Primers used in expression analysis of ER stress markers. (DOCX 2241 kb) [file 12885_2016_2611_MOESM1_ESM.docx]

**Additional file 1.**

**Baumann et al: Palmitate-induced ER stress increases trastuzumab sensitivity in HER2/neu-positive breast cancer cells**.

**Supplementary figure S1: Addition of exogenous palmitate does not increase cellular permeability of SKBR3 cells.**


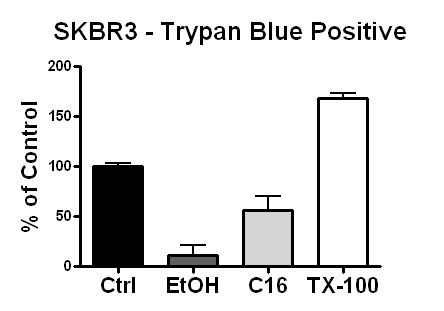


SKBR3 cells were treated with vehicle control (EtOH) or 250μM sodium palmitate for 24 hours. A 30 minute treatment of Triton X-100 (TX-100) served as a positive control [1]. Cells were harvested and treated with trypan blue and analyzed for cellular incorporation of the dye [2]. Data are normalized to untreated cells (Ctrl) and presented as means ± SD.

**Supplementary figure S2: Detailed results of the GO-term enrichment analysis for MCF7 cells using the DAVID Bioinformatics resource.**

The list of significantly altered transcripts was divided into two groups based on up- or downregulation after palmitate treatment and separately subjected to GO-term enrichment analysis. Results were obtained using the “Functional Annotation Chart" function of DAVID. The graph shows raw p-values, no association was significant after Bonferroni correction.

**Supplementary figure S3: Detailed results of the GO-term enrichment analysis for SKBR3 cells using the DAVID Bioinformatics resource.**

The list of significantly altered transcripts was divided into two groups based on up- or downregulation after palmitate treatment and separately subjected to GO-term enrichment analysis. Results were obtained using the “Functional Annotation Chart" function of DAVID. The graph shows Bonferroni-corrected p-values.

**Supplementary figure S4: Enrichment map illustrating gene member overlap in the enriched GO-terms for SKBR3 cells treated with palmitate.**

The map shows up- (red nodes, green edges) and downregulated (grey nodes, blue edges) GO-terms in SKBR3 cells. Nodes that include upregulated as well as downregulated genes are shown in pink. Nodes size corresponds to the number of genes that are associated with this particular GO-term. Edge distance indicates the Pearson correlation coeffcient between the nodes, whereas edge thickness indicates the degree of overlap (Jaccard-coeffcient). Input parameters: p-value < 0.005, FDR q-value < 0.1, Jaccard coeffcient < 0.25. Complete map is shown in the center; blown-up regions show groups of interest that scored with a high enrichment score in the Functional Annotation Clustering analysis.

**Supplementary figure S5: Gene set enrichment analysis of significantly altered transcripts in SKBR3 cells after palmitate treatment.**

A ranked list consisting of significantly up- and downregulated genes (p *·* 0.05) in SKBR3 cells after palmitate treatment was used as input for Gene Set Enrichment Analysis (GSEA) against the C2:curated gene sets in the MSigDB collection. Enrichment plots for the most significant overlap for the upregulated (left) as well as downregulated (right) genes are shown. The tables list the leading edge genes that contribute most to the computed overlap. The genes that show increased expression after palmitate treatment overlap with a set of genes upregulated after treatment with epoxomicin, a proteasome inhibitor (FDR q-value: 0.000, FWER p-value: 0.000). Genes that show decreased expression after palmitate treatment show significant overlap with a set of genes that are downregulated after growth factor deprivation (FDR q-value: 0.000, FWER p-value: 0.000).

**Supplementary figure S6: Exogenous palmitate does not alter the proteasome activity in HER2/neu-positive SKBR3 breast cancer cells.**

A. SKBR3 cells were transfected with the ZsProSensor-1 reporter construct which utilizes the ZsGreen1 fluorescent protein coupled to a proteasome-targeting sequence. Cells were treated with 250 µM palmitate, vehicle control or 0.5 µM of the proteasome inhibitor MG132. B. Fluorescent cells were counted after 24 h using the INCell Analyzer 2200.

**Supplementary figure S7: Expression of ER stress markers in HER2/neu-positive breast cancer cells upon treatment with palmitate.**


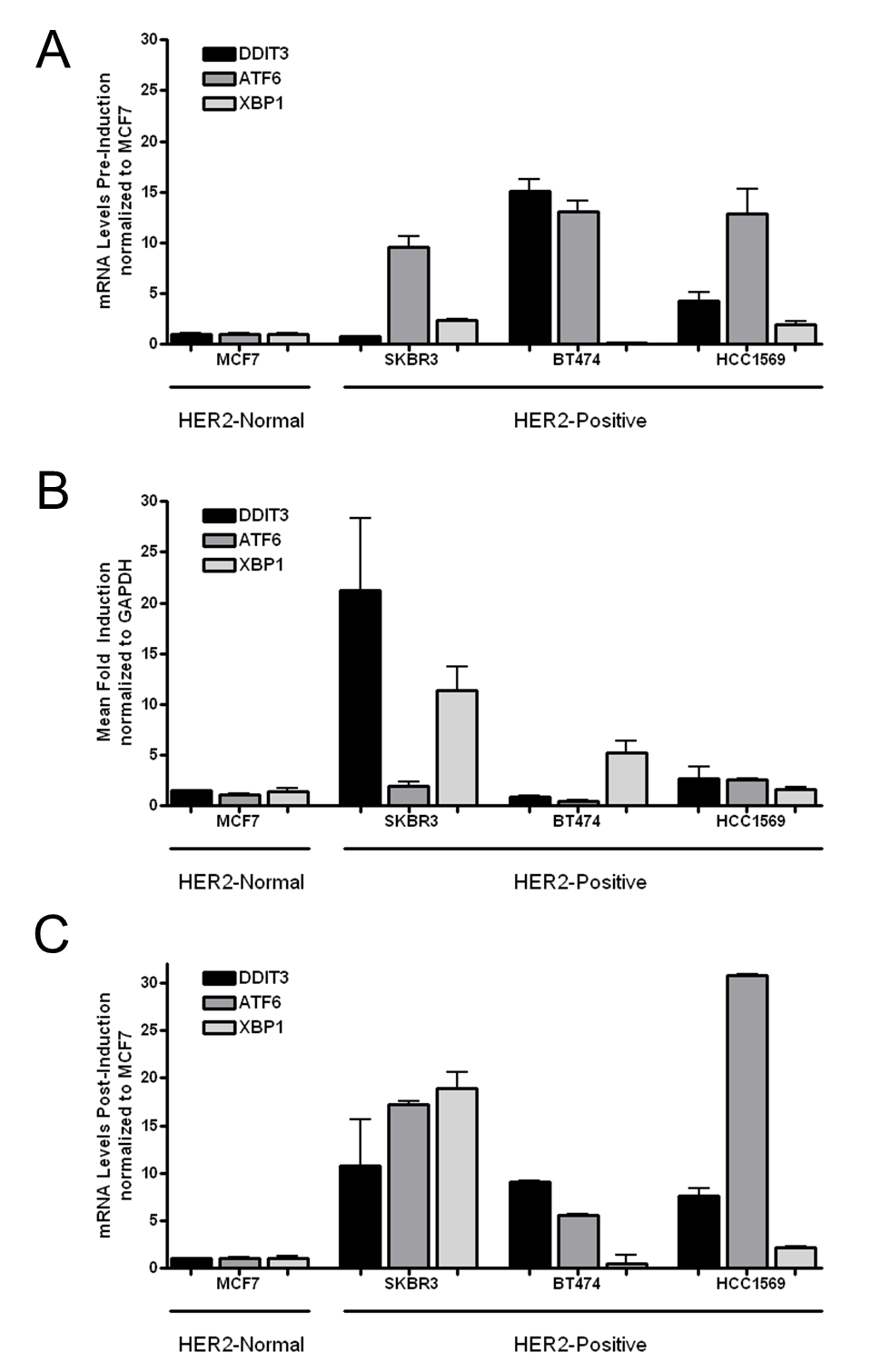


(**A**) mRNA level of DDIT3, ATF6 and XBP1 spliced transcripts treated with vehicle control. Data are normalized to MCF7 levels. (**B**) Cells are treated with 500μM sodium palmitate for 24 hours, and mean fold induction of the ER stress markers are determined. mRNA levels are relative to GAPDH levels and compare palmitate treated to vehicle control. (**C**) mRNA levels of ER stress markers on palmitate treated cells, normalized to MCF7 levels. Samples were run in triplicate and error bars represent the SEM of three biological replicates.

**Supplementary figure S8: Alterations of ER stress regulators associate with alterations of regulators of the lipogenic phenotype previously described in HER2/neu-positive breast cancer cell lines.**


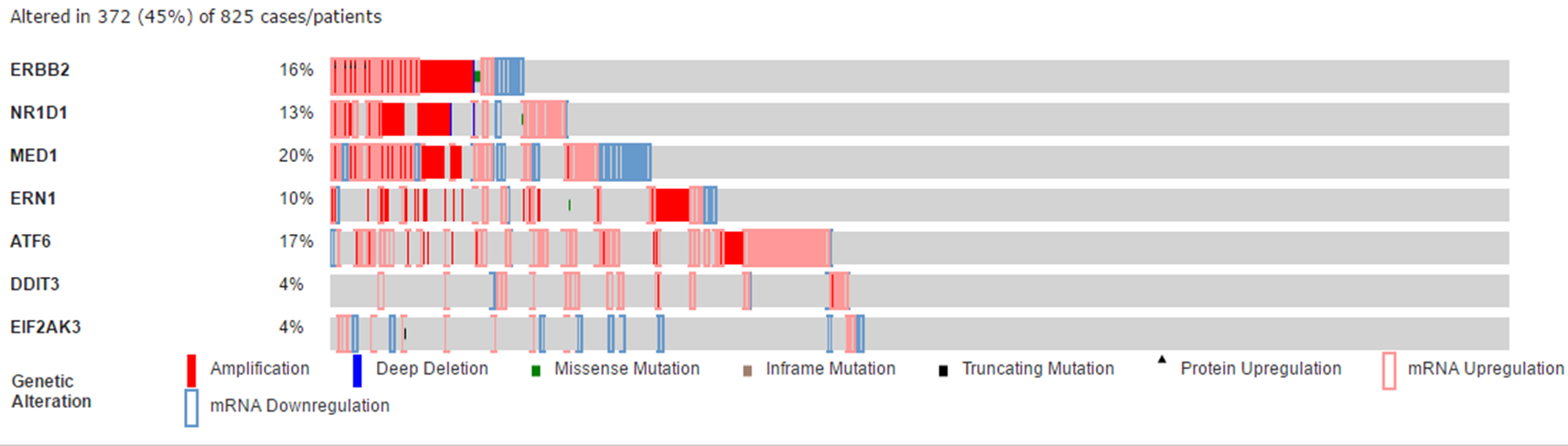


16%

13%

20%

10%

17%

4%

4%

HER2/neu

NR1D1

PBP

IRE1

ATF6

DDIT3/ CHOP

PERK

A

B

Increased expression of ER stress markers is associated with HER2/neu-positive breast cancer markers in human tumor sets. Molecular profiling data of 825 human breast tumors from the TCGA Breast Invasive Carcinoma project (http://cancergenome.nih.gov/). (**A**) Oncoprint displaying mutations and altered expression of genes related to the lipogenic phenotype of HER2/neu positive breast cancer (HER2/neu, NR1D1 and PBP) as well as several markers of ER stress network (IRE1, PERK, ATF6, DDIT3/CHOP). Individual tumor samples are shown on the X axis; relevant gene information on the Y axis. Mutation and altered expression is as shown in the key. (**B**) Analysis of mutual exclusivity and co-occurrence indicate statistically significant levels of co-occurrence of increased expression of ER stress markers in tumors with HER2/neu-positive lipogenic signatures.

**Supplementary figure S9: Her2/neu expression by itself has relatively minor effects on ER stress.**

**
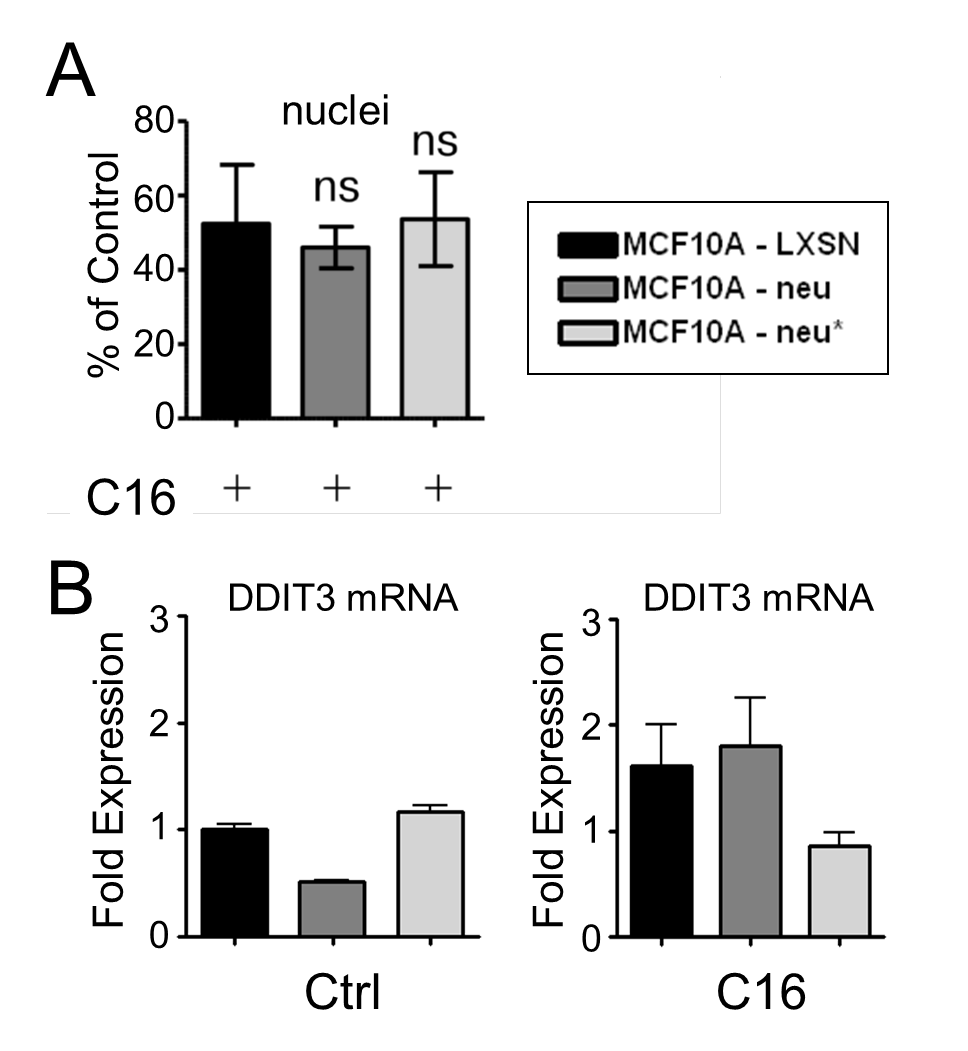
**

Effect of ectopic expression of Neu on the response to palmitate. Stable cell lines were generated using lentiviral vectors to overexpress normal Neu or constitutively active Neu*. Cells were then treated for 48h with 250 uM palmitate. **A**. Nuclei were counted and normalized to vehicle control. **B**. Basal levels of DDIT3 in MCF10A-neu, MCF10A-neu* cells normalized to MCF10A-LXSN. Cells were treated with 250 *μ*M palmitate or vehicle for 24 h and expression of DDIT3 assessed by qPCR. Error bars represent the SEM of three biological replicates.

Overexpression of HER2/neu in MCF10A breast epithelial cells does not induce sensitivity to palmitate. Treatment with palmitate slightly increased DDIT3 levels in MCF10A-neu cells, while decreased DDIT3 levels in MCF10A-neu* cells compared to MCF10A LXSN. However the magnitude of the changes is considerably smaller than seen in the HER2/neu positive lines. These results are consistent with previous studies that have established that the metabolic phenotype in “HER2/Neu-positive” cells is largely due to a constellation of other genetic alterations commonly found in this tumor type.

**Supplementary Table 1: Primers used in expression analysis of ER stress markers:**

| Target | Forward | Reverse |
| --- | --- | --- |
| ATF6[1] | 5’-TTGACATTTTTGGTCTTGTGG-3’ | 5’-GCAGAAGGGGAGACACATTT-3’ |
| DDIT3[1] | 5’-AGCCAAAATCAGAGCTGGAA-3’ | 5’-TGGATCAGTCTGGAAAAGCA-3’ |
| ERBB2[2] | 5’-CTCCTCCTCGCCCTCTTG-3’ | 5’-AGCATGTCCAGGTGGGTCT-3’ |
| ERBB3[2] | 5’-CCTGGACTTTCTGATCACCG-3’ | 5’-CCGTACTGTCCGGAAGACAT-3’ |
| XBP1 spliced[3] | 5’-CGAATGAGTGAGCTGGAACA-3’ | 5’-GGCCATGAGTTTTCTCTCGT-3’ |
| GAPDH[4] | 5’-GCAAATTCCATGGCACCGT-3’ | 5’-TCGCCCCACTTGATTTTGG-3’ |

ATF6 is induced during ER stress which upregulates the expression of XBP1. IRE1 splices XBP1 mRNA to yield a transcription factor that mediates the ER stress response [5]. Therefore, we measured levels of XBP1 using specific primers for the spliced form.

**Supplementary references.**

1. Balakrishnan B, Sen D, Hareendran S, Roshini V, David S, Srivastava A, Jayandharan GR: **Activation of the cellular unfolded protein response by recombinant adeno-associated virus vectors**. *PLoS One* 2013, **8**(1):e53845.

2. Ho-Pun-Cheung A, Bascoul-Mollevi C, Assenat E, Boissiere-Michot F, Bibeau F, Cellier D, Ychou M, Lopez-Crapez E: **Reverse transcription-quantitative polymerase chain reaction: description of a RIN-based algorithm for accurate data normalization**. *BMC Mol Biol* 2009, **10**:31.

3. Lee JW, Park HS, Park SA, Ryu SH, Meng W, Jurgensmeier JM, Kurie JM, Hong WK, Boyer JL, Herbst RS *et al*: **A Novel Small-Molecule Inhibitor Targeting CREB-CBP Complex Possesses Anti-Cancer Effects along with Cell Cycle Regulation, Autophagy Suppression and Endoplasmic Reticulum Stress**. *PLoS One* 2015, **10**(4):e0122628.

4. Yoeli-Lerner M, Yiu GK, Rabinovitz I, Erhardt P, Jauliac S, Toker A: **Akt blocks breast cancer cell motility and invasion through the transcription factor NFAT**. *Mol Cell* 2005, **20**(4):539-550.

5. Yoshida H, Matsui T, Yamamoto A, Okada T, Mori K: **XBP1 mRNA is induced by ATF6 and spliced by IRE1 in response to ER stress to produce a highly active transcription factor**. *Cell* 2001, **107**(7):881-891.
